# Megaprosthesis for a Rare Case of Bone Metastasis of Thyroid Carcinoma: Case Management and Surgical Approach

**DOI:** 10.7759/cureus.53717

**Published:** 2024-02-06

**Authors:** Mihnea-Alexandru Petre, Chrysoula Antoniadou, Mihai Emanuel Gherghe, Cristian Scheau, Serban Dragosloveanu

**Affiliations:** 1 Department of Orthopaedics, “Foisor” Clinical Hospital of Orthopaedics, Traumatology, and Osteoarticular TB, Bucharest, ROU; 2 Department of Pathology, “Foisor” Clinical Hospital of Orthopaedics, Traumatology, and Osteoarticular TB, Bucharest, ROU; 3 Department of Radiology and Medical Imaging, “Foisor” Clinical Hospital of Orthopaedics, Traumatology, and Osteoarticular TB, Bucharest, ROU

**Keywords:** management, pathology, imaging, bone metastasis, thyroid carcinoma, hürthle cell carcinoma

## Abstract

We present the case of a 56-year-old male patient diagnosed with Hürthle cell carcinoma (HCC) that developed widespread metastasis in bone, lung, and lymph nodes with a larger tumor located in the right tibia. The patient was only disturbed by the pain, discomfort, and disability linked to the tibial metastasis. After careful consideration, the best course of action was considered to be the surgical excision of the proximal right tibia with arthroplasty using the C LINK Megaprosthesis tumor revision system. Histological and immunohistochemical analyses were conducted on the tibial resection specimen. In addition, a comprehensive review of prior histological specimens from the primary thyroid tumor, lymph nodes, and lung was undertaken to evaluate the prognosis and provide guidance for the postoperative management of the patient.

## Introduction

Hürthle cell carcinoma (HCC), or oncocytic/oxyphilic cell carcinoma, has been recognized as an aggressive type of thyroid cancer and used to be classified as a variant of follicular thyroid tumors. Nevertheless, in the 2017 World Health Organization (WHO) Classification, HCCs are identified as a special type of tumor derived from thyroid follicles, distinguished from thyroid follicular tumors, and represent 3%-4% of thyroid carcinoma cases. From a therapeutic standpoint, there is no consensus on the optimal treatment method for HCC. The postoperative effect of radioactive iodine radiotherapy is unclear, as HCC has a lower ability to incorporate iodine compared to other thyroid carcinomas, and is therefore less responsive to this therapeutic approach [1-3]. Preoperative cytological, clinical, and genetic studies have not been shown to reliably discriminate between malignant and benign variants of Hürthle cell neoplasms (HCNs) [4]. Therefore, histopathological analysis remains the gold standard for diagnosing cases of HCC of the thyroid gland. This case report presents a patient diagnosed with thyroid carcinoma with bone metastasis, who underwent treatment in October 2023. The intervention involved using a knee MEGAsystem-cemented prosthesis for the knee joint, along with additional resection and reconstruction of the proximal tibia encompassing the bone tumor. The histopathological examination confirmed negative resection margins.

## Case presentation

**Figure 1:**
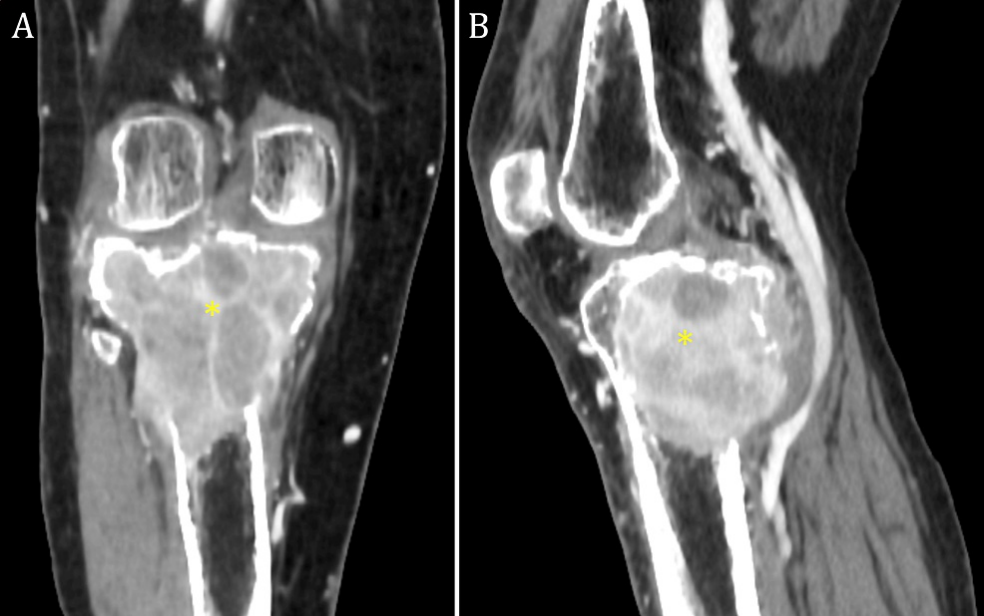
Preoperative contrast-enhanced computed tomography of the right knee: multiplanar reconstructions in the (A) coronal and (B) sagittal planes. Large lesion (*) in the proximal tibial epiphysis expanding past the cortical bone with a heterogeneous appearance, including necrotic and contrast-enhancing solid areas.

A 56-year-old male patient was referred to our service due to enduring left leg pain, discomfort, and a persistent inability to walk for three months. Since 2006, the patient had a history of thyroid nodules, with no family history of thyroid carcinoma. The patient was properly investigated in the following years, with routine imaging examinations such as computed tomography (CT), thyroid ultrasound, and scintigraphy, as well as thyroid hormone level assessment. On June 3, 2021, the patient presented to the oncology department, reporting left-side cervical and supraclavicular adenopathy for the last six months. The patient underwent an ultrasound that indicated a cervical mass, which raised a differential diagnosis between thyroglossal duct cyst, lymphadenopathy, lymphangioma, and thyroid tumor [5]. The patient then underwent a head, neck, and chest CT, which determined multiple enlarged lymph nodes in the left supraclavicular fossa, left cervical side, mediastinum, and multiple pulmonary masses. As the primary tumor origin was not yet assessed, various diagnoses were considered that included a primary thyroid cancer with multiple metastases as well as a primary lung cancer with multiple metastases [6]. A biopsy of a lymph node was conducted and the subsequent histopathological examination revealed a neoplasm with Hürthle cells. The biopsy did not include normal tissue, making it unclear if the specimen originated from the thyroid or if it was a secondary metastatic lesion in the lymph node. The patient received care from a multidisciplinary team consisting of specialists in oncology, radiotherapy, endocrinology, and thoracic surgery. The primary treatment for HCC consists of total thyroidectomy in all cases, with or without accompanying lymphadenectomy, and postoperative radioiodine therapy for all patients [7,8]. Total thyroidectomy along with cervical, mediastinal, left supraclavicular lymphadenectomy, and pulmonary metastesectomy were performed. The surgical specimens were submitted for pathological examination and the final histopathological diagnosis on the surgical specimen was poorly differentiated thyroid carcinoma with Hürthle cell features (oncocytic cell). The pTNM stage was pT3b pN1b pM1, with lymphovascular invasion (LVI) PN1 R1 stage II. Although the international literature does not present a consensus regarding the role of radioactive iodine therapy in HCC, with up to 40% not receiving this type of treatment [9-11], radioactive iodine radiotherapy was administered to the patient after the surgical procedures. The patient upheld multiple follow-up appointments and investigations in the meantime; hence, the oncology board affirmed his oncologic status had entered remission. On May 16, 2022, he complained of pain, discomfort, and difficulty walking; therefore, a CT scan of the lower limb was performed. The scan revealed an osteolytic lesion of the proximal right tibia, approx. 60/40 mm in size. The patient was referred to our hospital, where various imaging exams were conducted (Figure 1). Subsequently, the best course of action was considered to be the surgical excision of the proximal 13 cm of the right tibia with arthroplasty using the C Link Megaprosthesis tumor revision system. Proper digital planning was performed using dedicated software to ensure component size and fit [12].

Surgical technique

**Figure 2:**
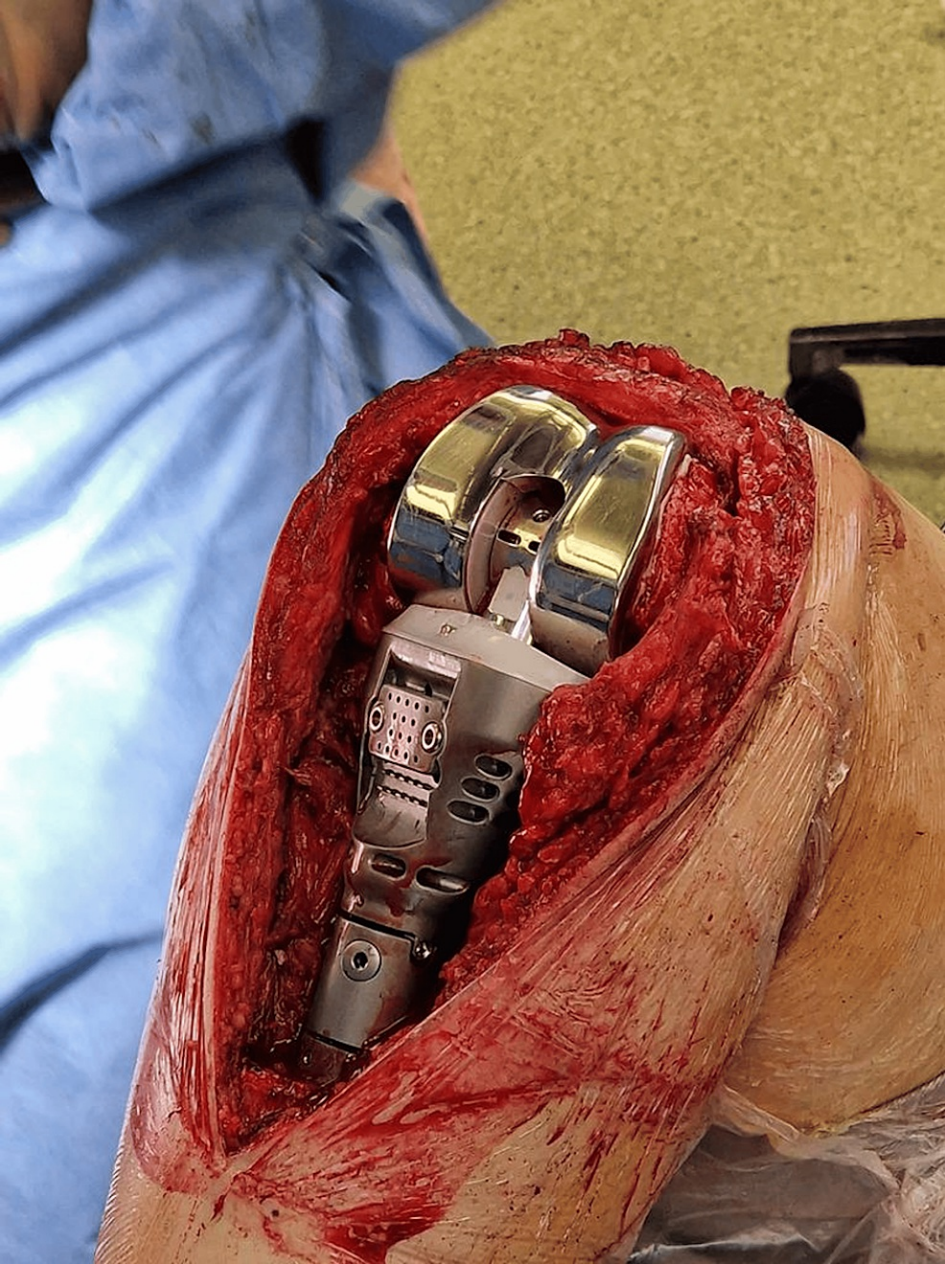
Knee Megaprosthesis System C Link intraoperative image.

The surgery was performed under general anesthesia, combined with an ultrasound-guided femoral nerve block. A tourniquet was placed but not inflated. A standard midline skin incision and extended medial parapatellar approach were used, with disinsertion of the extensor system. Careful dissection close to the bone was performed, with close attention to the posterior tibial neurovascular structures. The bone saw was used for cutting the proximal tibia below the calculated resection line. The next step was preparing the femur as described in the surgical technique guide provided by LINK. For the proximal tibia reconstruction implant, an extension stem was used, as shown in Figure 2.

**Figure 3:**
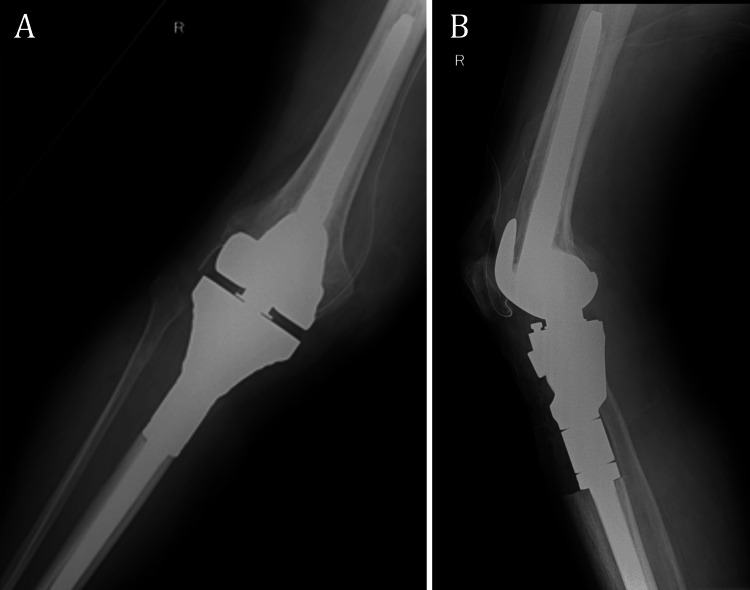
Postoperative radiographs of the right knee: (A) anteroposterior view; (B) lateral view.

After component trial and validation were performed, the final components were cemented and the extensor system was reinserted into the retentive tibial system. The wound closure was conducted with caution and one drainage tube was used for postoperative removal of blood. The patient was instructed to take enoxaparin 40 mg once a day for 30 days for postoperative chemoprophylaxis against venous thromboembolism. Following the surgical procedure, radiographic images of the knee were obtained as a routine postoperative check-up (Figure 3). Clinical and radiologic evaluations were performed at two weeks, six weeks, three months, six months, and one year after the surgery.

Histopathology

**Figure 4:**
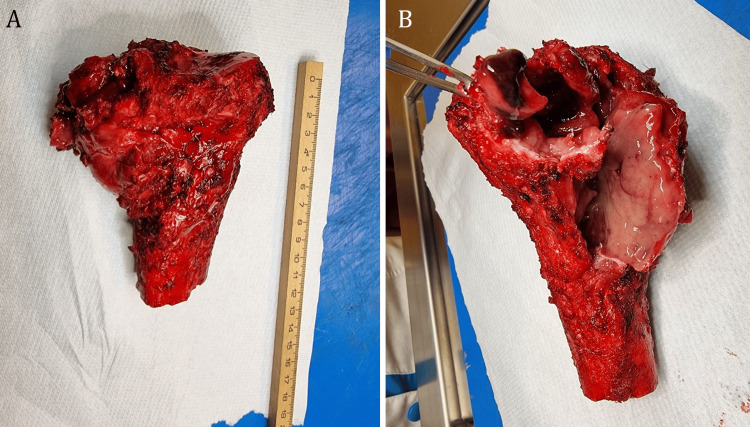
(A) Surgical specimen, 13 cm long, depicting the proximal tibia containing a large tumor, which appears to be entirely excised; (B) a large, hemorrhagic, and friable bone tumor.

The surgical specimen consisting of the resection of the proximal tibia was sent to the pathology department with the diagnosis of a bone malignant tumor, probably secondary to thyroid carcinoma. Macroscopically the specimen was a 13 cm proximal tibia entirely covered by periosteum but with a soft aria of complete destruction of the cortical bone (Figure 4).

**Figure 5:**
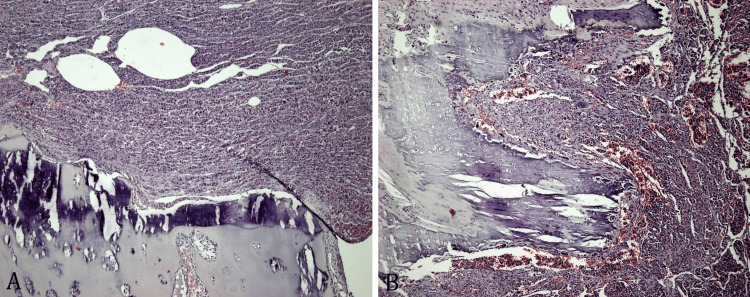
Hematoxylin and eosin (H&E), x4: (A) poorly differentiated thyroid carcinoma with oncocytic features invading the subchondral bone; (B) bone metastasis of poorly differentiated thyroid carcinoma with many vascular channels harboring tumor emboli.

Frozen sections were made and metastatic thyroid carcinoma was confirmed. Subsequently, the entire specimen was processed in 28 blocks of paraffin including the surgical bone resection margin, articular cartilage, and cortical bone to evaluate the extension of the tumor in the soft tissue, articular cavity, and surgical resection margins since the surgical procedure was intended to be radical. All histology slides showed malignant proliferation with solid, trabecular, insular, and limited follicular growth patterns consisting of large round and oval cells, with ample eosinophilic granular cytoplasm (oncocytic cells) and large nuclei with prominent nucleolus representing more than 75% of the tumor. Large hemorrhagic zones, foci of tumor necrosis, small cell components, and numerous tumor emboli were also present. Bone residual trabeculae indicating infiltrative growth were found at the edges of the surgical specimen (Figure 5).

**Figure 6:**
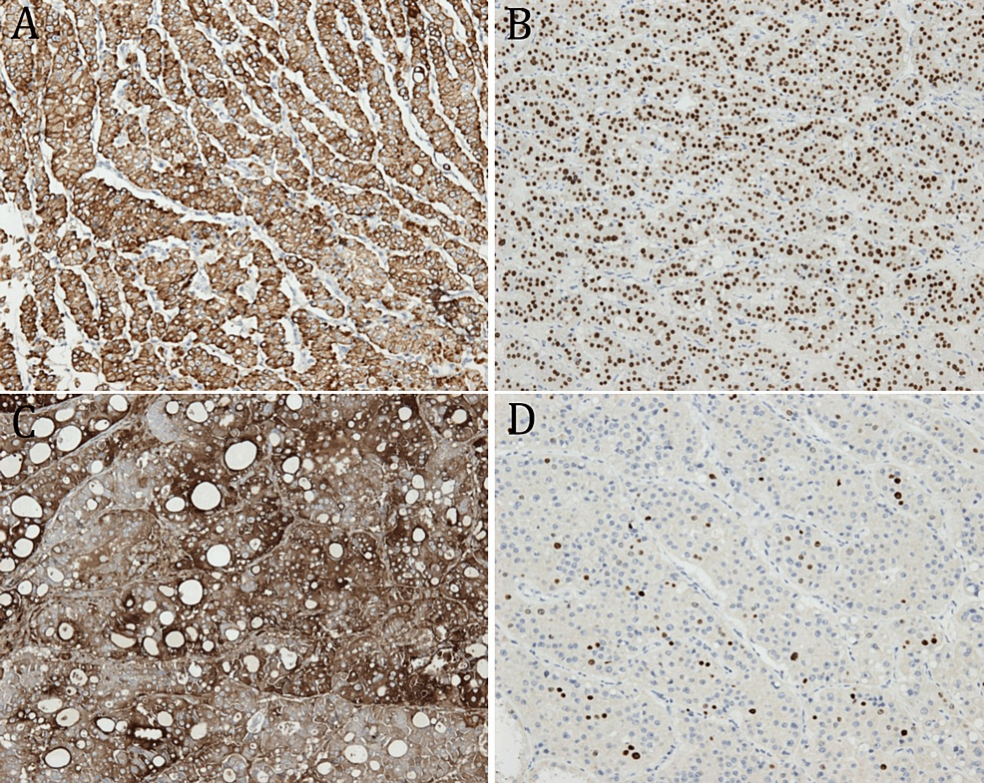
Immunohistochemistry (IHC), x4: (A) cytokeratin 19 intense cytoplasmic positivity in tumor cells; (B) thyroid transcription factor 1 nuclear diffuse positivity in tumor cells; (C) thyroglobulin diffuse cytoplasmic positivity in tumor cells; (D) Ki-67 proliferation index is approximately 15% in this field.

Immunohistochemistry (IHC) was performed using Leica Bond antibodies CD56 (clone 564), Ki-67 (clone MM1), thyroid transcription factor 1 (TTF1) (clone SPT24), and cytokeratin 19 (CK19) (clone b170) on the LEICA BOND III automatic machine. Thyroglobulin (thyroglobulin cocktail, mouse monoclonal 2H11 + 6E1) was performed manually. The findings were as follows: TTF1 nuclear intense positivity in the tumor, CK19 diffusely positive in the tumor, thyroglobulin diffusely positive in the cytoplasm of tumoral cells, Ki-67 mitotic index around 15%, and CD56-positive membranous in tumoral cells (Figure 6).

Based on the histological findings and immunohistochemical features, we concluded with the diagnosis of poorly differentiated oncocytic (Hürthle) cell carcinoma metastatic to the bone with free surgical margins.

**Figure 7:**
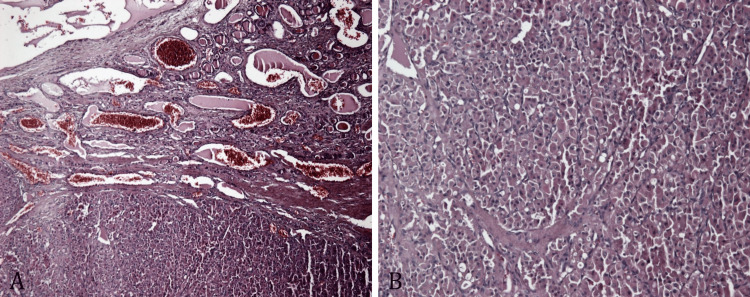
Hematoxylin and eosin (H&E), x10: (A) thyroid gland partially replaced by a poorly differentiated carcinoma with prevailing oncocytic features; (B) thyroid carcinoma consisting of large round and oval cells, with ample eosinophilic granular cytoplasm (oncocytic cells) and large nuclei with prominent nucleolus, showing a solid/trabecular growth pattern.

Retracing the patient history, we verified whether the tumor histology of the metastatic sites differed from the primary thyroid carcinoma (Figure 7). On the whole, while small differences were recorded, the histological pattern was similar to the findings in the resected bone specimen.

## Discussion

Historically, thyroid carcinomas have been divided into four types: papillary, follicular, medullary, and anaplastic [13,14]. HCC was considered a subtype of follicular thyroid carcinoma. However, in 2017, HCC, also known as oncocytic cell carcinoma of the thyroid, was classified as a distinct tumor type considering the significant histological, clinical, and prognostic differences of the tumor [14]. It was defined by most pathologists as an invasive, follicular-derived malignant neoplasm with >75% oncocytic cells [3,15].

The prognosis of HCC is believed to be related to the extent of vascular invasion, with the prognosis inversely correlating with the number of tumor-invaded veins. In large HCCs (>4 cm), foci of tumor necrosis, numerous mitoses (including abnormal forms), and foci of small tumor cells are prominent; these tumors have recently been identified as a subcategory of HCC: poorly differentiated HCC [3]. Furthermore, the Turin criteria that define the poorly differentiated thyroid carcinomas based on the presence of solid/trabecular/insular growth pattern, the absence of conventional nuclear features of papillary carcinoma, and the presence of either convoluted nuclei, mitotic activity >=3/10 high-power field (HPF) or tumor necrosis, can also be applied to poorly differentiated oncocytic thyroid carcinoma [16]. Some studies show that after adequate surgical treatment, oncocytic cell carcinoma has an encouraging prognosis, with rare relapses [17]. Unfortunately, the variation in survival statistics and reported metastatic potential suggests that the definitions of these lesions used in many reported series are not consistent [3]. Regarding the metastases in thyroid carcinomas, osseous metastases occur in only ~4% of all thyroid cancer patients and are consequently poorly studied but associated with considerably increased morbidity and mortality [18].

Looking at the patient history, the initial diagnosis on the thyroidectomy surgical specimen was “poorly differentiated thyroid carcinoma with oncocytic features applied also to the lung and lymph node metastasis.” Our findings on the bone metastasis were that more than 75% of the tumor is composed of oncocytic cells and trabecular, solid, and insular growth patterns were prevalent with rare follicular formations. Foci of small cells in the tumor and numerous vascular tumor emboli were also found. Based on this morphology and the Turin criteria [16] we concluded with the diagnosis of “poorly differentiated Hürthle cell (oncocytic) carcinoma.” Taking into consideration that the boundary between poorly differentiated HCC and poorly differentiated thyroid carcinoma with oncocytic features from the histological and IHC points of view are rather blurred, we cannot speculate on the relative prognosis of the above-mentioned entities. It is well known that oncocytic carcinomas of the thyroid are resistant to radioactive iodine ablation, a fact that explains the multiple metastases [3,16]. This fact does not necessarily correlate with a worse prognosis as long as the metastatic tumor can be removed entirely [17]. Even though data on this topic is scarce, some studies [19] regarding the prognosis of these thyroid carcinoma entities suggest a slightly favorable prognosis for the poorly differentiated carcinoma with oncocytic features over poorly differentiated HCC. The excellent physical and mental condition of the patient justified the surgical attitude to remove the metastatic tumor as a primary one, using the tumor prothesis implantation therefore providing the patient with the required autonomy. Restoring the knee’s anatomical features and function can be achieved through careful planning, selection of the adequate prosthesis type, and using an appropriate approach and surgical technique [20,21]. While extensive procedures may be associated with vascular or nervous complications, the need for bone allografts, or the addition of various biomaterials to compensate for intraoperative incidents [22], this was not the case in our patient. All risks were carefully considered and mitigated through thorough perioperative planning.

## Conclusions

HCC is a relatively rare subtype of thyroid carcinoma. Thyroid carcinoma with oncocytic features is a controversial subject in histopathology, with different subtypes of thyroid carcinomas presenting at least a component of oncocytic cells (Hürthle cells). In this patient, our study of all specimens showed no special differences regarding the histologic differentiation from the primary tumor to the metastatic sites. As such, other criteria than histological ones should be also taken into account in the management of bone metastases, and the clinical factors were favored over pathology features in this case. Personalized patient treatment should be employed after thorough case discussion and judgment in a multidisciplinary board. When the risk of relapse is evaluated as reasonable and all patient-related factors are considered, improvements in the quality of life can be paramount.
